# STIM2 protects hippocampal mushroom spines from amyloid synaptotoxicity

**DOI:** 10.1186/s13024-015-0034-7

**Published:** 2015-08-15

**Authors:** Elena Popugaeva, Ekaterina Pchitskaya, Anastasiya Speshilova, Sergey Alexandrov, Hua Zhang, Olga Vlasova, Ilya Bezprozvanny

**Affiliations:** Laboratory of Molecular Neurodegeneration, Department of Medical Physics, Peter the Great St.Petersburg Polytechnic University, St. Petersburg, Russian Federation; Laboratory of Microscopy and Microanalysis, Department of Physics-chemistry and Microsystem Technique, Institute of Metallurgy, Mechanical Engineering and Transport, Peter the Great St.Petersburg Polytechnic University, St. Petersburg, Russian Federation; Department of Physiology, UT Southwestern Medical Center at Dallas, Dallas, TX 75390 USA

**Keywords:** Alzheimer disease, Abeta peptides, STIM2, Mushroom spines, Synapse

## Abstract

**Background:**

Alzheimer disease (AD) is a disease of lost memories. Mushroom postsynaptic spines play a key role in memory storage, and loss of mushroom spines has been proposed to be linked to memory loss in AD. Generation of amyloidogenic peptides and accumulation of amyloid plaques is one of the pathological hallmarks of AD. It is important to evaluate effects of amyloid on stability of mushroom spines.

**Results:**

In this study we used *in vitro* and *in vivo* models of amyloid synaptotoxicity to investigate effects of amyloid peptides on hippocampal mushroom spines. We discovered that application of Aβ42 oligomers to hippocampal cultures or injection of Aβ42 oligomers directly into hippocampal region resulted in reduction of mushroom spines and activity of synaptic calcium-calmodulin-dependent kinase II (CaMKII). We further discovered that expression of STIM2 protein rescued CaMKII activity and protected mushroom spines from amyloid toxicity *in vitro* and *in vivo*.

**Conclusions:**

Obtained results suggest that downregulation of STIM2-dependent stability of mushroom spines and reduction in activity of synaptic CaMKII is a mechanism of hippocampal synaptic loss in AD model of amyloid synaptotoxicity and that modulators/activators of this pathway may have a potential therapeutic value for treatment of AD.

**Electronic supplementary material:**

The online version of this article (doi:10.1186/s13024-015-0034-7) contains supplementary material, which is available to authorized users.

## Background

Alzheimer disease (AD) is the most common reason for elderly dementia in the world, and its prevalence will continue to increase with the aging population. The search for effective AD therapies is an urgent need. Many brain regions such as temporal lobe, parietal lobe, frontal cortex and etc. are damaged in AD. Among them hippocampus is the best studied region. Hippocampus associated memory loss in AD results from “synaptic failure” [1–3]. The synapse is formed by presynaptic axon ending and by postsynaptic dendritic spine. There are three morphological groups of dendritic spines: mushroom, stubby and thin spines [4, 5]. It has been proposed that mushroom spines are stable “memory spines” that make functionally stronger synapses and therefore responsible for memory storage [6]. Thin spines are suggested to be “learning spines” since they response to increase and decrease in synaptic activity [6]. We and others previously proposed that hippocampal mushroom spines are strongly eliminated in AD and that loss of mushroom spines may underlie cognitive decline during the progression of the disease [7–11]. However, cell biological mechanisms responsible for loss of mushroom spines in AD are poorly understood.

In the recent study we demonstrated that neuronal store-operated calcium entry (nSOC) in postsynaptic spines play a key role in stability of mushroom spines [10]. We further demonstrated that synaptic nSOC is controlled by stromal interaction molecule 2 (STIM2) and that STIM2-nSOC pathway is downregulated in hippocampal neurons from the mutant mice containing M146V familial AD mutation in presenilin 1 (PS1-M146V-KI) [10]. Moreover, we have demonstrated that expression of STIM2 protein rescues synaptic nSOC and mushroom spine loss in PS1-M146V-KI hippocampal neurons [10]. However, the PS1-M146V-KI mice do not express human amyloid precursor protein (APP) and do not generate human Aβ peptides, which believed to be one of the key components of pathogenic process in AD [1, 3, 12]. The goal of the present study is to evaluate effects of human Aβ peptides on stability of mushroom spines. By using *in vitro* and *in vivo* models of amyloid synaptotoxicity we concluded that downregulation of STIM2-dependent stability of mushroom spines is a mechanism of synaptic loss in AD model of amyloid synaptotoxicity and that modulators/activators of this pathway may have a potential therapeutic value for treatment of memory loss in AD.

## Results

### Amyloid oligomers destabilize mushroom spines in primary hippocampal neuronal cultures

**Fig. 1:**
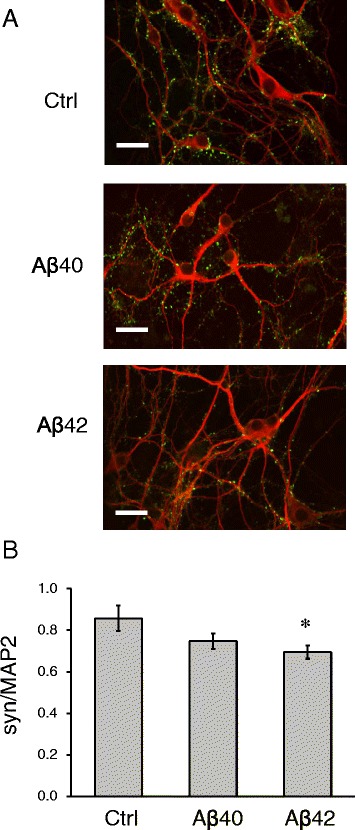
Synaptotoxic effects of amyloid oligomers in primary neuronal cultures. **a** Primary hippocampal neuronal cultures exposed to Aβ40, Aβ42 or vehicle treated (Ctrl). The cultures were fixed and stained for synaptic marker Synapsin I (green) and neuronal marker MAP2 (red). Scale bar corresponds to 20 μm. **b** Mean fluorescent intensities of synapsin I staining were divided by mean fluorescent intensities of MAP2 staining for hippocampal cultures treated with Aβ40, Aβ42 or vehicle treated (Ctrl). Average results from three independent experiments are shown. Values are shown as mean ± SEM. *: *p* < 0.05 by ANOVA one-way test

In our experiments we set out to establish neuronal culture model of amyloid toxicity. In these studies we focused on soluble amyloid oligomers, which have been demonstrated to exert synaptotoxic effects in AD [1, 12–17]. Soluble amyloid oligomers were prepared from synthetic Aβ42 peptides by following previously described procedures [18] (for details see Materials and Methods section). The oligomeric state of resulting Aβ42 preparation was confirmed by AFM analyses as well as by SDS gel Western blotting experiments (Additional file 1: Figure S1). According to AFM data Aβ42 oligomers appear as globular structures with 1–2 nm height and around 10 nm width (Additional file 1: Figure S1A). Western blot experiments showed that Aβ42 preparation is mainly in oligomeric form and its molecular weight is around 26 kDa (Additional file 1: Figure S1B) that corresponds to pentamers or hexamers [19]. To mimic physiological situation more closely, we utilized low concentrations (less than 100 nM as calculated based on initial amount of peptides utilized for preparation of oligomers) of Aβ42 oligomers in our experiments. We also evaluated effects of Aβ40 oligomers. Aβ40 peptides were processed the same way as Aβ42 peptides (Additional file 1: Figure S1A). Equivalent amounts of Aβ42 and Aβ40 peptides were used in our experiments. Generated oligomers of Aβ42 and Aβ40 were added to DIV11 primary hippocampal neuronal cultures. In control experiments neuronal cultures were treated with vehicle (equivalent amount of growth media). At DIV14 these cultures were fixed, permeabilized and stained for neuronal marker MAP2 and synaptic marker synapsin I (Fig. 1a). To evaluate synaptogenesis state in studied cultures, mean synapsin signal intensity was divided by the mean of MAP2 intensity. Consistent with previous observations [15, 16, 20], we observed significant loss of synapsin staining in cultures exposed to Aβ42 oligomers (Fig. 1a, 1b). There was also a trend towards synaptic loss in cultures exposed to Aβ40 oligomers, but these changes have not reached a level of significance when compared to control cultures (Fig. 1a, 1b).

**Fig. 2:**
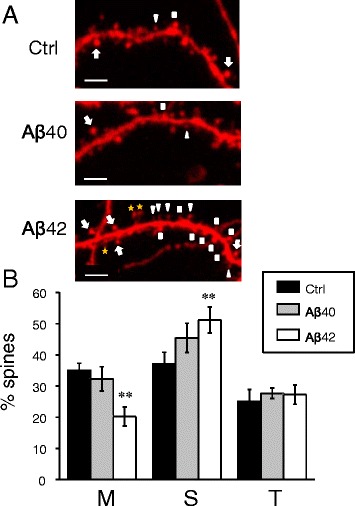
Amyloid oligomers cause loss of hippocampal mushroom spines *in vitro.* **a** Primary hippocampal neurons from WT mice were transfected with TD-Tomato and visualized by confocal imaging. Representative images are shown for cultures exposed to Aβ40, Aβ42 or vehicle (Ctrl). Scale bar corresponds to 5 μm. On the Aβ42 panel all quantified types of spines are indicated: MS with an arrow, T with a triangle, S with a square, dendritic elements that were not counted as spines are labelled with a yellow star. The mean data from four independent experiments are shown. **b** Average percentages of mushroom (M), stubby (S) and thin (T) spines are shown for cultures exposed to Aβ40, Aβ42 or vehicle (Ctrl). For spine quantification *n* = 8–10 (for each treatment per one experiment) neurons were analyzed. Values are shown as mean ± SEM. **: *p* < 0.005 by ANOVA one-way and post hoc tests

Previous studies demonstrated a shift from mushroom to stubby synaptic spines in organotypic hippocampal slice preparation from APP_SDL_ transgenic mice [21]. To analyse how synaptic spine shape is affected by application of soluble amyloid oligomers, we adopted spine morphology analysis approach that was used in our previous study with PS1-M146V KI cultures [10]. In these experiments primary hippocampal cultures were transfected at DIV7 with plasmid expressing TD-Tomato fluorescent protein. On DIV11 cells were treated with Aβ40 or Aβ42 oligomers or vehicle treated. The neurons were fixed at DIV14, imaged by confocal microscopy and spine shapes were automatically analysed by a software (see Methods for details). Consistent with the previous report [10], mushroom spines constituted 35 % of total spines in control (vehicle treated) cultures at DIV 14 (Fig. 2a, 2b). Addition of Aβ40 oligomers had no significant effect on mushroom spines in neuronal cultures (Fig. 2a, 2b). In contrast, addition of Aβ42 oligomers resulted in significant reduction in mushroom spine proportion (Fig. 2a). On average, the fraction of mushroom spines was reduced to 20 % in cultures treated with Aβ42 oligomers (Fig. 2b). Following treatment with Aβ42, the fraction of stubby spines was increased from 35 to 50 % and the fraction of thin spines remained constant at 26 % (Fig. 2b). The shift from mushroom towards stubby spines induced by application of Aβ42 oligomers is consistent with the previous results obtained in organotypic hippocampal slices from APP_SLD_ transgenic model [21]. This is in contrast with our previous studies with neurons from the PS1-M146V KI mice, where we observed the shift between mushroom and thin spines [10].

### STIM2 protein is downregulated in response to *in vitro* application of amyloid oligomers

**Fig. 3:**
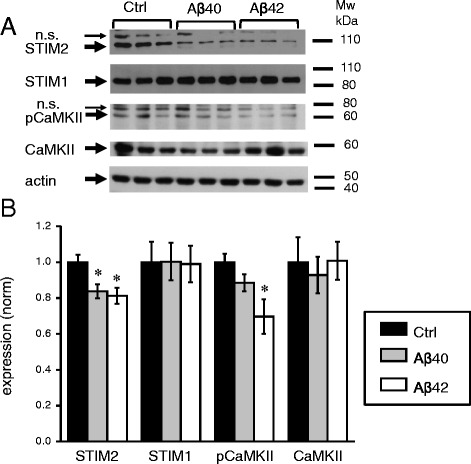
STIM2 and pCaMKII are downregulated in hippocampal cultures treated with amyloid. **a** The expression levels of STIM2, STIM1, pCaMKII, and CaMKII were analyzed by Western blotting of lysates from WT hippocampal cultures exposed to Aβ40, Aβ42 or vehicle (Ctrl). Actin was used as a loading control. **b** Quantification of STIM2, STIM1, pCaMKII and CaMKII expression levels in WT cultures exposed to Aβ40, Aβ42 or vehicle (Ctrl) (normalized to actin levels). Graph represents the data from three independent experiments. Values are shown as mean ± SEM. *: *p* < 0.05 by ANOVA one-way and post hoc tests, n.s. (non specific)

In our recent publication [10] we demonstrated that stability of mushroom spines depends on STIM2-mediated neuronal store-operated calcium influx (nSOC) and continuous activity of Ca^2+^ /calmodulin-dependent protein kinase II (CaMKII) which is enriched in synaptic spines. To find out if this pathway affected by application of amyloid oligomers we performed a series of Western blotting experiments with lysates prepared from control cultures and the cultures treated with Aβ40 and Aβ42 oligomers. In these experiments we observed approximately 20 % reduction in STIM2 expression levels in Aβ40 and Aβ42 treated cultures (Fig. 3a, 3b). We also observed 30 % reduction of auto-phosphorylated form of CaMKII (pCaMKII) in Aβ42-treated cultures (Fig. 3a, 3b). There was a trend to reduction of pCaMKII levels in Aβ40-treated cells, but it did not reach statistical significance (Fig. 3a, 3b). The levels of STIM1 protein and total CaMKII levels were not affected in Aβ40 or Aβ42-treated cultures (Fig. 3a, 3b). Notably, selective reduction in STIM2 expression and reduced levels of pCaMKII are similar to changes observed in our previous experiments with PS1-M146V-KI neurons [10], suggesting that common signalling pathways are affected in synaptic spines in both cellular models of AD pathology. However, changes in PS1-M146V-KI neurons were more dramatic than in Aβ42-treated neurons, with 75 % reduction in STIM2 levels and 40 % reduction in pCaMKII signal [10].

### STIM2 overexpression protects mushroom spines from synaptotoxic effects of amyloid oligomers *in vitro*

**Fig. 4:**
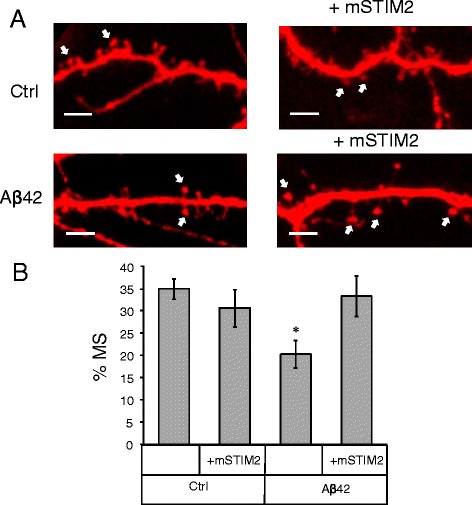
STIM2 overexpression protects mushroom spines from amyloid toxicity *in vitro.* **a** Primary hippocampal cultures were co-transfected with TD-Tomato and mSTIM2 plasmids or transfected with TD-Tomato. After transfection cells were treated with Aβ42 oligomers or vehicle (Ctrl). Spine morphology was visualized by confocal imaging. Scale bar corresponds to 5 μm. Mushroom spines are indicated with an arrow. **b** Percentages of mushroom spines (MS) in hippocampal cultures co-transfected with TD-Tomato and mSTIM2 or transfected with TD-Tomato. Data collected from the three batches of cultures are shown for vehicle treated cultures (Ctrl) and cultures treated with Aβ42 oligomers. For spine quantification *n* = 6–8 (for each treatment per one experiment) neurons were analyzed. Experiment was repeated three times. Values are shown as mean ± SEM. *: *p* < 0.05 by *t*-test

In the previous publication [10] we demonstrated that expression of STIM2 protein and upregulation of synaptic nSOC pathway results in rescue of mushroom spines in PS1-M146V-KI neurons. We supposed that the same approach could protect mushroom spines from amyloid toxicity. In the next series of experiments we co-transfected hippocampal neurons at DIV7 with TD-Tomato plasmid and the plasmid encoding mouse STIM2 (mSTIM2). On DIV11 the cells were treated with Aβ42 oligomers or vehicle treated. The neurons were fixed at DIV14 and morphology of the spines was visualized by confocal imaging (Fig. 4a). Consistent with the previous findings (Fig. 2), we discovered that application of Aβ42 oligomers resulted in significant reduction in a fraction of mushroom spines in hippocampal neurons (Fig. 4a, 4b). Expression of STIM2 had no significant effect on the fraction of mushroom spines in control cultures, but resulted in complete rescue of mushroom spine loss in Aβ42-treated cultures (Fig. 4a, 4b). From these results we concluded that expression of STIM2 is able to protect hippocampal mushroom spines from synaptotoxic effects of Aβ42.

### Injection of Aβ42 oligomers causes loss of hippocampal mushroom spines *in vivo*

**Fig. 5:**
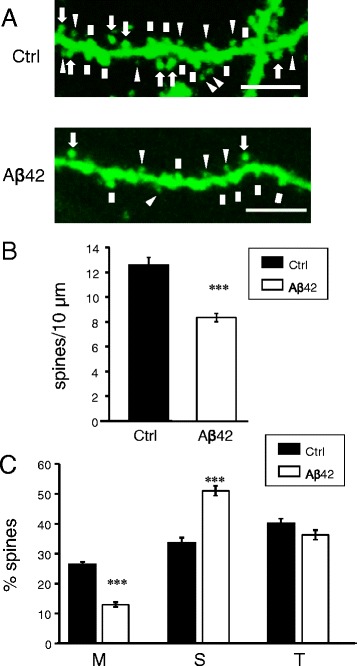
Influence of Aβ42 on mushroom spines *in vivo*. **a** The spine shape of CA1 hippocampal neurons from the 3.5 months old line M mice injected with Aβ42 and control mice was visualized by two-photon imaging. Scale bar corresponds to 5 μm. On the Ctrl panel all quantified types of spines are indicated: M (mushroom) with an arrow, T (thin) with a triangle and S (stubby) with a square. **b**, **c** Total spine density and percentages of mushroom, thin and stubby spines in neurons from CA1 hippocampal area. Graph represents the data of three independent experiments with control mice and the mice injected with Aβ42. For spine quantification *n* = 21–30 neurons were analyzed. Values are shown as mean ± SEM. ***: *p* < 0.0005 by *t*-test

Do amyloid oligomers result in loss of mushroom spines *in vivo*? To answer this question, we performed stereotaxic injections of synthetic Aβ42 oligomers to CA1 region of 2 months old line Thy1-GFP line M mice [22]. In control AFM analyses and SDS gel Western blotting experiments we confirmed formation of oligomeric Aβ42 species in these preparations (Additional file 1: Figure S1A, B). The stock concentrations of Aβ42 injection solution in these experiments was equal to 1 μM based on the initial peptide content. Control line M mice were injected with secondary antibodies conjugated to Alexa-555 fluorophore. Six week after injections synaptic morphology in these mice was analysed by two-photon imaging performed with hippocampal slices (Fig. 5a). When obtained results were analysed, we discovered that injection of Aβ42 oligomers resulted in significant reduction in total spine density (Fig. 5b). We also found that the fraction of mushroom spines was significantly reduced and the fraction of stubby spines was significantly increased in CA1 (stratum radiatum) area of hippocampus of the mice injected with Aβ42 oligomers (Fig. 5c). The fraction of thin spines was not affected in these experiments (Fig. 5c). The shift from mushroom to stubby spines observed in these experiments in response to injection of Aβ42 oligomers is similar to results obtained in our primary culture experiments (Fig. 2) and in published reports with organotypic hippocampal slices [21].

### Injection of Aβ42 oligomers causes downregulation of STIM2 *in vivo*

**Fig. 6:**
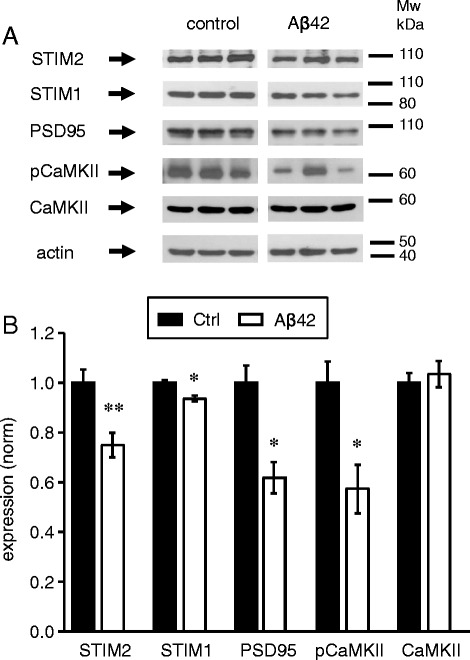
STIM2, PSD95 and pCaMKII are downregulated in mice injected with Aβ42. **a** The expression levels of STIM2, STIM1, PSD95, pCaMKII, CaMKII were analyzed by Western blotting of hippocampal lysates taken from wild type mice injected with Aβ42 and from the control mice. Each band on WB panel represents one mice. Actin was used as a loading control. **b** Expression of STIM2, STIM1, PSD95, pCaMKII, and CaMKII proteins was normalized to actin. Values are shown as mean ± SEM for control mice and the mice injected with Aβ42. *: *p* < 0.05, **: *p* < 0.005 by *t*-test

In order to investigate effects of Aβ42 injections *in vivo*, we performed Western blotting analyses of hippocampal lysates prepared from wild type mice injected with Aβ42 oligomers. Mice injected with the secondary antibody were used as a control. Consistent with hippocampal culture data (Fig. 3) we observed 20 % reduction of STIM2 levels in the hippocampus of the mice injected with Aβ42 oligomers (Fig. 6a, 6b). Also consistent with the culture data (Fig. 3), we observed 45 % reduction in the levels of pCaMKII following injection of Aβ42 oligomers (Fig. 6a, 6b). Levels of STIM1 protein were reduced by less than 5 % and the total levels of CaMKII were not affected in Aβ42-injected mice (Fig. 6a, 6b). We also evaluated levels of PSD95 protein, that is enriched in postsynaptic density of mushroom spines. Levels of PSD95 were also reduced by 40 % in hippocampus of Aβ42-injected mice (Fig. 6a, 6b). Significant reduction of PSD95 and pCaMKII levels in these experiments is consistent with the loss of mushroom spines in hippocampal neurons of the mice injected with Aβ42 oligomers (Fig. 5) and with our previous studies with PS1-M146V-KI mice [10].

### STIM2 overexpression protects mushroom spines from amyloid toxicity *in vivo*

**Fig. 7:**
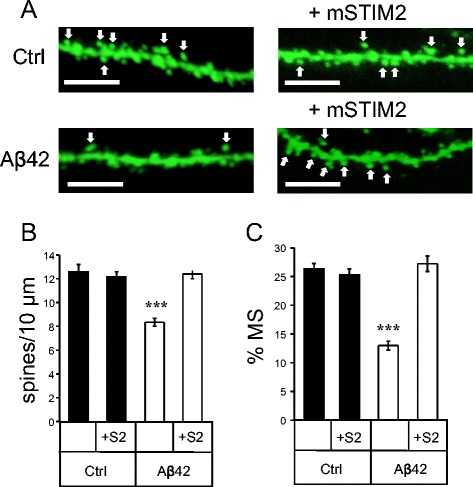
STIM2 overexpression protects mushroom spines from amyloid toxicity *in vivo*. **a** The spine shape of CA1 hippocampal neurons from 3.5 months old line M mice injected with AAV1-mSTIM2 (S2), Aβ42, control mice (Ctrl), and Aβ42 together with AAV1-mSTIM2 (S2) was visualized by two-photon imaging. Scale bar corresponds to 5 μm. Mushroom spines are indicated with an arrow. **b**, **c** Total spine density and percentages of mushroom spines (MS) in neurons from CA1 hippocampal area of mice injected with AAV1-mSTIM2 (S2), control mice, mice injected with Aβ42 and mice injected with Aβ42 together with AAV1-mSTIM2 (S2). Graph represents the data of three independent experiments. For spine quantification *n* = 21–30 neurons were analyzed. Values are shown as mean ± SEM. ***: *p* < 0.0005 by *t*-test

**Fig. 8:**
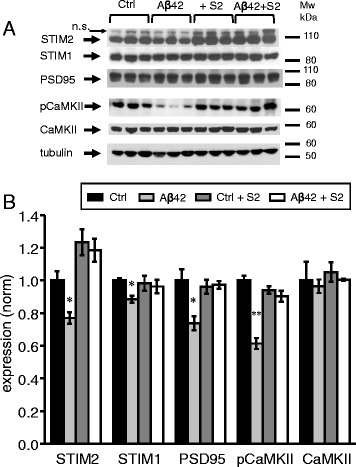
STIM2 overexpression rescues levels of PSD95 and pCAMKII proteins in conditions of amyloid toxicity *in vivo.* **a** The expression levels of STIM1, PSD95, pCaMKII, and CaMKII were analyzed by Western blotting of hippocampal lysates taken from 3.5 months old mice injected with AAV1-mSTIM2 (S2), Aβ42, control mice (Ctrl), and Aβ42 together with AAV1-mSTIM2 (S2). Each band on WB panel represents one mice. Tubulin was used as a loading control. **b** Expression of STIM1, PSD95, pCaMKII, CaMKII proteins was normalized to tubulin. Mean values from 3 independent experiments are shown as mean ± SEM for the mice injected with AAV1-mSTIM2 (S2), Aβ42, control mice (Ctrl), and Aβ42 together with AAV1-mSTIM2 (S2). *: *p* < 0.05, **: *p* < 0.005 by *t*-test

To evaluate potential neuroprotective effects of STIM2 expression *in vivo*, we performed stereotaxic injections of AAV1-mSTIM2 virus together with Aβ42 oligomers (at 1 μM concentration) to CA1 region of 2 months old Thy1-GFP line M mice. Control mice were injected with AAV1-mSTIM2 together with the secondary antibodies. Neuronal and synaptic morphology in CA1 area (stratum radiatum) was analysed by two-photon imaging with hippocampal brain slices six weeks after injection (Fig. 7a). Consistent with the previous findings (Fig. 5), we found that injection of Aβ42 oligomers results in significant loss of synaptic spines (Fig. 7b) and reduction in the fraction of mushroom spines (Fig. 7c). Expression of STIM2 protein had no effect on total spine density or the fraction of mushroom spines in control experiments, but resulted in complete rescue of total spine loss and mushroom spine loss induced by Aβ42 oligomers (Fig. 7b, 7c). To further confirm these findings we performed Western blotting of hippocampal lysates from these mice (Fig. 8). In these experiments we discovered that expression of STIM2 prevents reduction of PSD95 and pCaMKII levels in hippocampal region of Aβ42-injected mice (Fig. 8a, 8b).

## Discussion

### Loss of mushroom spines in cellular and animal models of AD

AD is a disease of lost memories. Studies in learning and memory field suggested that persistent increase in synaptic strength is used to achieve long-term modification of neuronal network properties and store memories. It has been proposed that the mushroom spines between excitatory neurons are stable “memory spines” that make functionally stronger synapses and therefore responsible for memory storage [6]. By taking this one step further, we and others previously proposed that loss of mushroom spines may underlie cognitive decline during the progression of the AD [7–11]. Recent experimental evidence in several cellular and animal models of AD provided support to this hypothesis. Fraction of mushroom spines was significantly reduced in hippocampal slice cultures from APP_SDL_ transgenic mice that express human APP695 with Swedish (KM595/596NL), Dutch (E618Q), and London (V642I) mutations under control of platelet-derived growth factor β promoter [21]. More subtle model of amyloid toxicity was generated recently. In this model human APP transgene with Dutch mutation (E693Q) was expressed under control of neuronal Thy1 promoter (DU mice) [23]. In contrast to most APP transgenic mice, DU mice generate soluble Aβ oligomers without formation of amyloid plaques [23]. Such phenotype mimics phenotype of patients with Arctic (E693G) and E693Δ mutations that show AD phenotype in the absence of fibrillar amyloid accumulation [24, 25]. Anatomical analysis of spines in CA1 area of hippocampus in DU mice revealed significant reduction in post-synaptic density (PSD) of mushroom spine synapses at 12 months of age [11]. In the present study we observed significant reduction in the fraction of mushroom spines in neuronal cultures exposed to Aβ42 oligomers (Fig. 2) and in CA1 area (stratum radiatum) of hippocampus of the mice injected with Aβ42 oligomers (Fig. 5). Although Price at al have not reported reduction in the fraction of mushroom spines in their analysis of DU mice [11], there was a trend to reduced mushroom spine density in their data (Fig. 3 in Price et al., 2014). Most likely concentration of Aβ42 oligomers in our study was higher than in the DU mice, leading to more dramatic destabilization of mushroom spines in our experiments. Interestingly, loss of mushroom spines is not unique to amyloid toxicity models. In the recent study we observed significant loss of mushroom spines in hippocampal neurons from PS1-M146V-KI mouse model [10]. Thus, loss of mushroom spines appear to be a common feature of AD models, in agreement with our hypothesis [7, 9].

Important to note that there is a correlation between dendritic spines alterations in CA1 area of hippocampus and memory impairments in different mice models of AD. Loss of mushroom spines in CA1 hippocampal neurons in PS1-M146V-KI [10] may underlie disrupted late-phase LTP and age-related alterations in hippocampal spatial memory observed in these mice [26]. Twelve month old DU mice have decreased PSD length and trend to reduced mushroom spine density in CA1 area as well as diminished performance in the water maze indicating that these mice exhibit perturbed hippocampus-associated spatial learning and memory [11]. It has been demonstrated that the Aβ-mediated impairment of memory in rats is associated with lower density of synapses and altered synaptic structure in both the dentate gyrus and CA1 fields [27].

### Synaptic CaMKII and stability of mushroom spines

What are the signalling pathways that maybe responsible for mushroom spine loss in AD? We previously proposed that stability of the mushroom spines is determined by a balance between activity of synaptic CaMKII and calcineurin [28]. Tackenberg at al (2009) demonstrated that inhibition of NMDAR rescues mushroom spine defect in APP_SDL_ organotypic slices [21]. They further argued that activation of postsynaptic calcineurin downstream of NMDAR leads to mushroom spine loss in APP_SDL_ slices in their experiments [21]. Price et al. (2014) did not dissect signalling pathways responsible for mushroom spine PSD shrinkage in DU mouse model [11]. Results observed in the current study suggest that activity of synaptic CaMKII has been reduced following application of Aβ42 oligomers. Indeed, we observed significant reduction in pCaMKII signals in hippocampal neurons following exposure to Aβ42 *in vitro* (Fig. 3) and *in vivo* (Fig. 6). These effects were specific for active synaptic CaMKII, as total levels of CaMKII were not affected by Aβ42 oligomers in these experiments (Figs. 2 and 6). These results are consistent with the previously reported loss of synaptic localization of CaMKII in cortical neurons from APP transgenic mice (APP Swedish mutation, KM670/671NL) or in cortical cultures exposed to Aβ peptides [29]. Similar reduction in pCaMKII levels was observed in our recent study in hippocampal neurons from PS1-M146V-KI mouse model [10]. Moreover, reduction in hippocampal pCaMKII levels was observed in our experiments as a part of normal aging process [10]. Thus, reduction of synaptic CaMKII activity appears to be a common feature of AD mouse models and probably relevant for aging-related memory impairment as well. Importantly, dysregulated phosphorylation of synaptic CaMKII was reported at MCI stage of human AD [30], suggesting that reduced activity of synaptic CaMKII may indeed contribute to human disease.

### Synaptic STIM2-nSOC pathway as novel therapeutic target for AD

It follows from the above discussion that activators of synaptic CaMKII should expert neuroprotective effects. Indeed, overexpression of CaMKII protected synaptic function in Aβ42-treated cortical neurons [29]. However, direct expression of CaMKII is not likely to become a viable therapeutic strategy for AD. In the previous study we discovered that activity of synaptic CaMKII is regulated via STIM2-nSOC Ca^2+^ influx pathway [10]. We further demonstrated that STIM2 levels are reduced in PS1-M146V-KI hippocampal neurons and that overexpression of STIM2 rescues pCaMKII activity and mushroom spines in this model of AD [10]. The disruption of STIM2-nSOC signalling pathway was also observed in recently developed APP-KI mouse model [31]. In the present study we discovered that STIM2 levels are reduced in neuronal cultures exposed to Aβ42 and Aβ40 oligomers (Fig. 3) and in hippocampus of the mice injected with Aβ42 oligomers (Fig. 6). Similar to study with PS1-M146V-KI mice, these effects were specific for STIM2 with minimal effect on STIM1 (Figs. 3 and 6). However, effects on STIM2 expression were less dramatic in conditions of amyloid toxicity (present study) than in experiments with PS1-M146V-KI neurons [10]. It appears that the downregulation of STIM2-nSOC pathway is a primary mechanism responsible for mushroom spine loss in conditions of ER Ca^2+^ dysregulation resulting from PS1-M146V-KI mutation [10, 32]. In contrast, Aβ42 oligomers more likely to act on other Ca^2+^-related postsynaptic targets such as NMDAR [33] and mGluR5 [20, 34], with downregulation of STIM2-nSOC pathway occurring as a secondary event. Interestingly that Aβ40 significantly reduced expression of STIM2 (Fig. 3) but didn’t significantly influence expression of pCaMKII (Fig. 3) and stability of mushroom spines (Fig. 2). We speculate that Aβ40 at nanomolar concentration doesn’t affect postsynaptic Ca^2+^-channels (such as mentioned above). Therefore, in our experimental conditions Aβ40 is not as toxic as Aβ42 and doesn’t cause reduction in pCaMKII and mushroom spines numbers.

Critically, we demonstrated that overexpression of STIM2 rescued mushroom spine loss in cultures exposed to Aβ42 oligomers (Fig. 4), rescued mushroom spine loss (Fig. 7) and pCaMKII levels (Fig. 8) in the hippocampus of the mice injected with Aβ42 oligomers. It appears that enhanced activity of STIM2-nSOC pathway was sufficient to rescue activity of synaptic CaMKII and protects mushroom spines in conditions of amyloid toxicity as well as in conditions of ER Ca^2+^ dysregulation due to PS1-M146V mutation. From these results we concluded that activators of STIM2-nSOC synaptic Ca^2+^ influx pathway may have a therapeutic value for treatment of AD.

## Conclusions

Obtained results lead us to conclude that loss of mushroom spines is a common feature of AD models and it is observed in presenilin mutant neurons [10] and in conditions of amyloid toxicity [8, 11, 31], (present study). We further conclude that reduction in synaptic activity of CaMKII is a likely cause of mushroom spine destabilization in AD models [10, 29, 31], (present study) and in human AD [30]. Expression of STIM2 protein rescued CaMKII activity and mushroom spine defects in presenilin model of AD [10] and in conditions of amyloid toxicity (present study). Thus, positive modulators of STIM2-nSOC may constitute potential therapeutic agents for treatment of AD and related pathologies.

## Methods

### Mice

Albino outbred mice (Rappolovo farm, Leningradsky District, Russia) were used as source of brain tissue for experiments with hippocampal cultures. Pilot culture experiments were performed with neurons from the FVB strain pups. Obtained results were similar to results obtained with albino outbred mice (data not shown). Therefore genetic background should not influence the reproducibility of neuronal culture experiments in this study. Line M mice Tg(Thy1-EGFP)MJrs/J [22] was used in imaging experiments. Line M mice were obtained from the Jackson Laboratory (Stock Number: 007788) and the breeding colony was established in the animal facility located in the Laboratory of Molecular Neurodegeneration in St Petersburg State Polytechnical University. Western blot analyses of hippocampal lysates were done using WT mice (C57BL/6 J background) obtained from the Jackson Laboratory (Stock Number: 000664). All procedures involving mice were approved by the Institutional Animal Care and Use Committee of the Research Institute of Influenza Ministry of healthcare of the Russian Federation, in accord with the Ministry of agriculture of the Russian Federation guidelines.

### Plasmids and viruses

pCSCMA-TD-tomato plasmid was purchased from Addgene. Generation of mSTIM2 expression plasmid and AAV1-mSTIM2 adeno-associated virus was previously described [10]. The high titer stock of AAV1-mSTIM2 virus (titer >10^13^ vp/ml) was obtained from the University of Iowa Gene Transfer Vector Core.

### Aβ40 and Aβ42 preparation

Aβ42 (#20276) and Aβ40 (#24236) peptides were purchased from AnaSpec (Fremont, USA). A lyophilized aliquot (1 mg) of Aβ42 and Aβ40 peptides was dissolved in 80 μl of 1 % NH_4_OH and then in 920 μl of sterile phosphate-buffered saline (PBS) to get stock solution with concentration 1 mg/ml (stored as 100 μl aliquots at – 20 °C). Working Aβ solutions were made immediately before treatment of the cells by diluting stock concentration to 0.1 μM final Aβ peptide concentrations in Neurobasal-A medium (Gibco, Life technologies, USA). Working solutions were incubated at + 4 °C 24 h to obtain the oligomeric conditions as described by Zheng et al. [18]. At the day of the usage working solutions were centrifuged at 14000 g, + 4 °C, 10 min to purify oligomeric Aβ fraction from fibrils. Composition of supernatant fraction was confirmed by atomic force microscopy (Additional file 1: Figure S1A) and by denaturating (0.1%SDS) 15 % gel electrophoresis followed by Western blot with anti-Aβ 6E10 monoclonal antibodies (Covance, SIG-39320) (Additional file 1: Figure S1B). Western blot was repeated three times. Amounts of Abeta loaded per gel lane are 1.2 nmol for Aβ42 and Aβ40 and 12 nmol for Aβ42-555. The supernatant fractions containing Aβ oligomers were used to treat hippocampal cultures. In experiments with stereotaxic injections Aβ42 peptides conjugated with Alexa-555 fluorophore were used (#60480-01, AnaSpec). Working solutions of Aβ42 peptides peptides for *in vivo* experiments were 1 μM. Other steps in preparation of working solution were the same as described above for *in vitro* experiments.

### Primary hippocampal cultures

The hippocampal cultures from albino outbred mice were established from postnatal day 0–2 pups and maintained in culture as we described previously [10, 32]. Both hippocampi from pups were dissected in sterile ice cold 1XHBSS buffer (pH 7.2). Hippocampi were dissociated in papain solution (Worthington 3176) at 37 °C, 30 min. To remove big undissociated cell aggregates solution with hippocampal neurons were twice triturated in 1 μg/ml DNAseI (Sigma, DN-25). To remove DNAseI neurons were centrifuged at 1500 rpm, 4 min. Supernatants discarded and fresh warm (37 °C) growth medium (Neurobasal-A (Gibco, 10888), 1xB27 (Gibco, 17504), 1 % heat inactivated FBS (Gibco, 16000), 0.5 mM L-Glutamine (Gibco, 25030)) was added. Neurons were plated in 24 well plate containing 12 mm round Menzel cover slip (d0-1) precoated with 1 % poly-D-lysine (Sigma, p-7886). Neurons were seeded at ~5×10^4^ cells per well (24 well format). Growth medium was changed on the next day after plating then weekly. In control experiments neuronal cultures were treated with equivalent amount (same volume as used to prepare Aβ solutions) of Neurobasal A incubated at + 4 °C 24 h (vehicle).

### Immunohistochemistry

Hippocampal primary neurons from albino outbred mice were fixed with fixation solution (4 % formaldehyde in PBS, pH 7.4) for 30 min at room temperature, washed three times with PBS with 0.05 % Tween-20 then incubated at room temperature for 1 h in 5 % BSA in PBS buffer. Primary antibodies anti-map2 mAb (1:1000, Chemicon, mAB378) and anti-synapsin I (1:1000, Chemicon, Temecula, CA) were diluted in 2.5 % BSA in PBS with 0.025 % Tween-20 and incubated at room temperature for 3 h. After three times wash, the hippocampal cultures were incubated in 2.5 % BSA in PBS solution with the secondary antibody (1:1000, Alexa Fluor 488 or 594, Invitrogen) for 1.5 h at room temperature and visualized by a confocal microscope (Thorlabs, Russia). In order to compare data from different culture experiments mean fluorescent intensity of Channel A (synapsin) was divided by the mean fluorescent intensity of Channel B (MAP2).

### Calcium-phosphate transfection of primary hippocampal cultures

Calcium-phosphate transfection of primary hippocampal cultures was done as previously described [10, 32]. Changes to published protocol were in following steps: transfection medium was prepared 4 days before transfection (to equilibrate osmolality of transfection medium with growth medium); at the day of transfection MgCl_2_ was added to the transfection medium to the final concentration 10 mM and sterile filtered mixtures of plasmids with CaCl_2_ were incubated during 5-7 min at room temperature. Calcium transfection kit was purchased from Sigma (CAPHOS).

### Dendritic spine analysis in primary hippocampal neural cultures

Dendritic spines can be classified into three types: mushroom, thin and stubby. However, sometimes spines of unknown shape such as spines with two heads, spines with common neck (Fig. 2a, Aβ42 sample) or spines with transitional states can be identified. For better understanding of spine analysis we have labelled all spines that were counted and dendritic segments that were not counted as spines in Fig. 2a. Different types of spines are indicated as following: MS (mushroom spine) with an arrow, T (thin) with an triangle and S (stubby) with square. Those spines that have not been characterized as mushroom, thin or stubby have been labelled with a yellow star (Fig. 2). For assessment of synapse morphology, hippocampal cultures were transfected with TD-tomato plasmid at DIV7 using the calcium phosphate method and fixed (4 % formaldehyde in PBS, pH 7.4) at DIV14. A Z-stack of optical section was captured using 100X objective (1.4 NA Olympus, UPlanSApo) with a confocal microscope (Thorlabs, USA). Each image maximal resolution was 1024 × 1024 pixels with 0.1 μm/pixel and averaged six times. Total Z volume was 6 – 8 μm imaged with Z interval 0.2 μm. At least 18 transfected neurons for each treatment from three independent experiments were used for quantitative analysis. Quantitative analysis for dendritic spines was performed by using freely available NeuronStudio software package [35] as we previously described [10]. To classify the shape of neuronal spines in culture, we adapted an algorithm from published method [35]. In classification of spine shapes we used the following cutoff values: aspect ratio for thin spines (AR_thin(crit)) = 2.5, head to neck ratio (HNR(crit)) = 1.4, and head diameter (HD(crit)) = 0.45 μm, the Z interval was 0.2 μm. These values were defined and calculated exactly as described by previous report [35].

### Stereotaxic injection

The stereotaxic injections of AAV1-mSTIM2 virus or Aβ42 oligomers in CA1 area (stratum pyramidale) of hippocampus were performed as described previously [10]. Mice were injected with 5 ul of Aβ42-Alexa555 oligomer, prepared as described above. Additionally, 1 μl of AAV1-mSTIM2 virus was injected each side. Control mice were injected with 5 μl of secondary anti-mouse antibody Alexa-555 (Invitrogen, A11005) diluted in Neurobasal-A to the concentration 1 μM and incubated at + 4 °C 24 h. For bilateral hippocampus injection, the Hamilton injection syringes were positioned at a 10 ° angle on both sides and the optimal injection coordinates (from bregma) were anterior/posterior (AP) -2.0 mm, lateral +2.6 mm, dorsal/ventral-1.9 mm. 1 μl of virus was injected each side. For STIM2 overexpression, 12 Thy1-GFP and 12 WT mice were injected at 8 weeks of age, 3 mice for each experimental group.

### Western blot analyses

Hippocampal regions from WT mice at 3.5 month were dissected, homogenized, and solubilized at 4 °C for 1 h in lysis buffer (1 % CHAPS, 137 mM NaCl, 2.7 mM KCl, 4.3 mM Na2HPO4, 1.4 mM KH2PO4, pH 7.2, 5 mM EDTA, 5 mM EGTA, 1 mM PMSF, 50 mM NaF, 1 mM Na3VO4 and protease inhibitors). Primary hippocampal neurons were collected, homogenized and solubilized in the same buffer on DIV14. The total protein lysates were separated by SDS-PAGE and analyzed by Western blotting with anti-STIM1 pAb (1:1000, Cell Signaling, 4916 s), anti-STIM2 pAb (1:1000, AnaSpec, 54681), anti-Phospho-CaMKII (1:2000, Abcam, ab171095), anti-CaMKII (1:1000, Chemicon, MAB8699), anti-PSD95 (1:1000, Cell Signaling, 3450 s), anti-actin clone C4 (1:1000, Millipore, MAB1501) and anti-tubulin (1:1000, DSHB, E7-c). HRP-conjugated anti-rabbit (1:2000, DAKO, P0448) and anti-mouse (1:2000, DAKO, P0447) secondary antibodies. Analysis of the data was performed using Quantity One from BioRad software. The mean density of each band was normalized to actin or tubulin signal in the same sample and averaged. All Western blots replicated 3–7 times.

### Dendritic spine analysis in mice hippocampus

Six weeks after virus injection Thy1-GFP mice were anesthetized with 200 μl of 250 mg/ml Urethane (Sigma, U2500-250G), then intracardially perfused with ice cold 1.5 % paraformaldehyde (PFA) solution in phosphate buffer (pH 7.4) 30 ml in 3 min. The brains were extracted and post-fixed in 1.5 % PFA solutions for 16 h before cutting. Thick (300 μm) hippocampal sections from the fixed brains were obtained using vibratome (WPI, NVSLM1, USA) and floated in 0.5 % PFA solution at 4 °C. Brain slices analyzed by two-photon imaging (Thorlabs, USA) with 20X lens (1 NA Olympus, XLUMPlanFl N). The Z interval was 0.5 μm. Aβ42 after injection are spread along CA1 area (Additional file 2: Figure S2). In mice injected with Aβ42 and control mice injected with Alexa555-labeled antibody, with or without AAV-mSTIM2, the secondary apical dendrites of hippocampal CA1 pyramidal neurons were selected for taking images. Each image was acquired at 1024 × 1024 pixels with the pixel size 0.065 μm and averaged twelve times. Seven to nine neurons were analyzed per animal, approximately 20–30 neurons from 3 mice for each group. To classify the shape of neuronal spines in slices we used NeuronStudio software package and an algorithm from [35] with the following cutoff values: AR_thin(crit) = 2.5, HNR(crit) = 1.3, HD(crit) = 0.2 μm. Acquisition of the images as well as morphometric quantification was performed by ‘blinded’ operators.

### Statistical analyses

The results are presented as mean ± SEM. Statistical comparisons of results obtained in experiments were performed by Student’s *t* test for two-group comparisons. ANOVA one way and post hot test were used for comparison of data from more than two groups. The statistical method and p values are indicated in figure legends as appropriate.

## Additional files

Additional file 1: Figure S1. Characterization of oligomeric state of Aβ42, Aβ40 and Aβ42-Alexa555. (A) Atomic force microscopy (performed as described in Additonal file 3) images of Aβ42, Aβ40 and Aβ42-Alexa555 samples after 24 h incubation at 4 °C visualized on graphite. Sizes of field of view for left images are 4×4μm and for right images 1×1μm. Topographic profile for each Aβ sample is presented (measured globule is marked with blue line). All Aβ samples appear primarily as globular structures with following sizes: Aβ42 height 1.56 ± 0.3 nm, diameter at fwhm before deconvolution 75 ± 0.6 nm, diameter at fwhm after deconvolution 9.6 ± 0.1 nm; Aβ40 height 0.9 ± 0.1 nm, diameter at fwhm before deconvolution 43 ± 0.3 nm, diameter at fwhm after deconvolution 7.3 ± 0.1 nm; Aβ42-Alexa555 height 1.46 ± 0.2 nm, diameter at fwhm before deconvolution 68 ± 0.6 nm, diameter at fwhm after deconvolution 8.9 ± 0.1 nm. (B) Supernatant fractions of Aβ42, Aβ40 and Aβ42-Alexa555 preparations were separated on 15 % Acrylamide/Bis SDS gel and analyzed by Western blotting with anti-Aβ 6E10 monolconal antibodies. (PDF 375 kb) Additional file 2: Figure S2. Visualization of hippocampal neurons injected with Alexa-555 labelled Aβ42. Low-magnification image of CA1 hippocampal area from 3.5 months old Thy1-GFP line M mouse is presented. Image is taken six weeks after injection. CA1 neurons expressing GFP protein are shown in green, signals from Alexa-555 labeled Aβ42 are shown in red. The specific dendritic segment where the data on spine density and shape were obtained is marked with white circle. Scale bar corresponds to 20 μm. (PDF 51 kb) Additional file 3:
**Supplementary experimental procedure.** Atomic force microscopy. Imaging was performed on commercial SPM Solver P47-Pro atomic force microscope with NSG 11 probe (Nt-MDT Co., Zelenograd, Moscow, Russia). Images were taken in air using tapping mode on highly ordered pyrolytic graphite (Nt-MDT Co., Zelenograd, Moscow, Russia). The surface of graphite was carefully rinsed with deionized water and gently dried under a N_2_ stream. NovaRC1 operating software version 850 was used to acquire the data images, Gwyddion and SPIP software were used to render the data and perform the analyses. The z-height of 9-10 globules from two different areas on the graphite was measured. Particle analysis was performed by choosing a threshold height equivalent to 1/2 the average z-height of the globules. This measures globule diameter at fwhm (full width at half-maximum). To get approximate width of globules geometrical deconvolution model [36] was used, particles were treated as spheres $$ \mathrm{h}=\raisebox{1ex}{$\sqrt{w/2}$}\!\left/ \!\raisebox{-1ex}{$2Rt$}\right. $$ where h is the real width of the structure, w is the width or diameter observed in the AFM image, and Rt is the tip apex radius (in our case it is around 10 nm). (DOCX 12 kb)
