# Supplementary figures and images for: STIM2 protects hippocampal mushroom spines from amyloid synaptotoxicity
Source: Mol Neurodegener. 2015 Aug 15;10:37. doi: 10.1186/s13024-015-0034-7 (PMC4536802; doi:10.1186/s13024-015-0034-7)

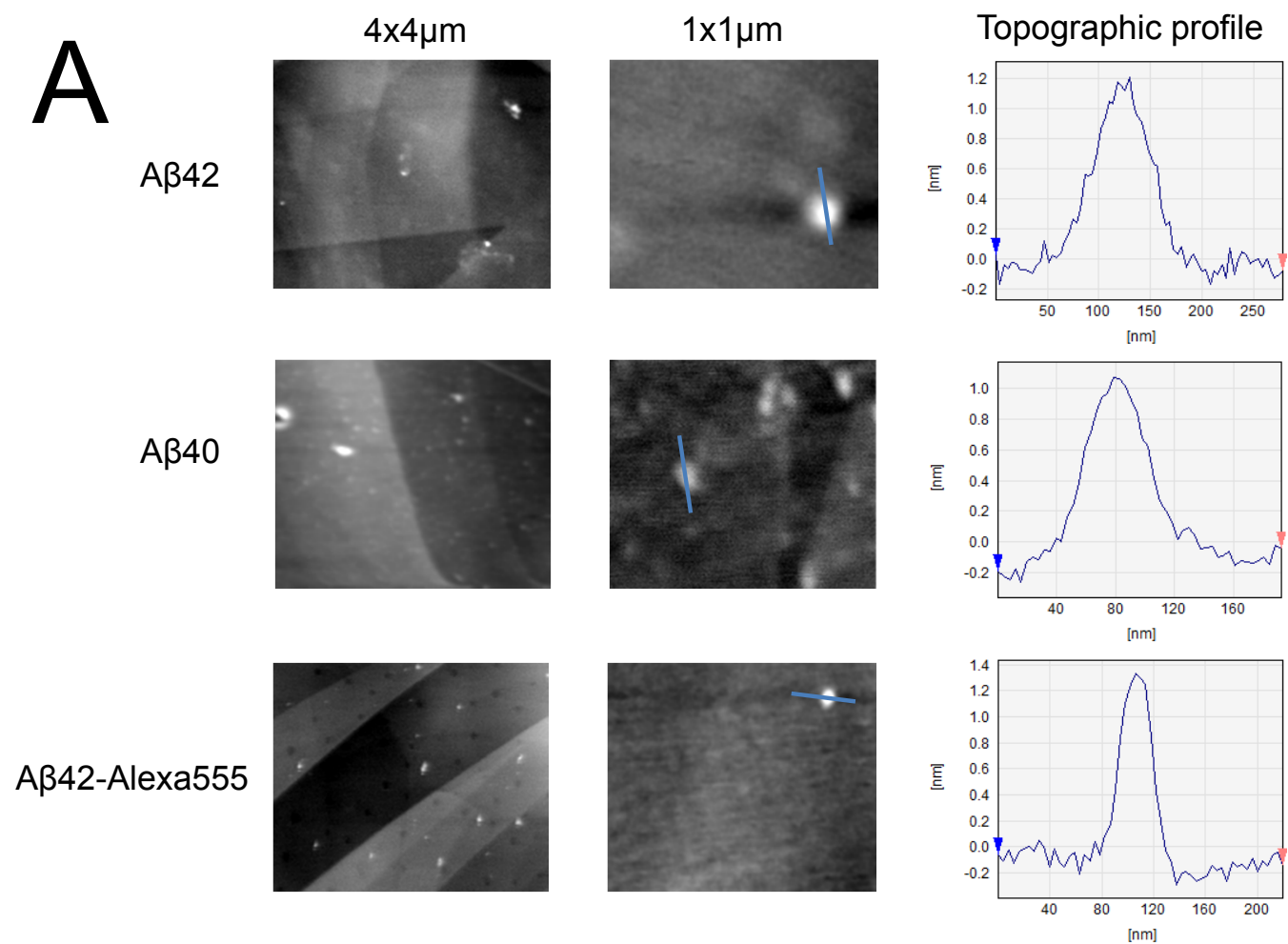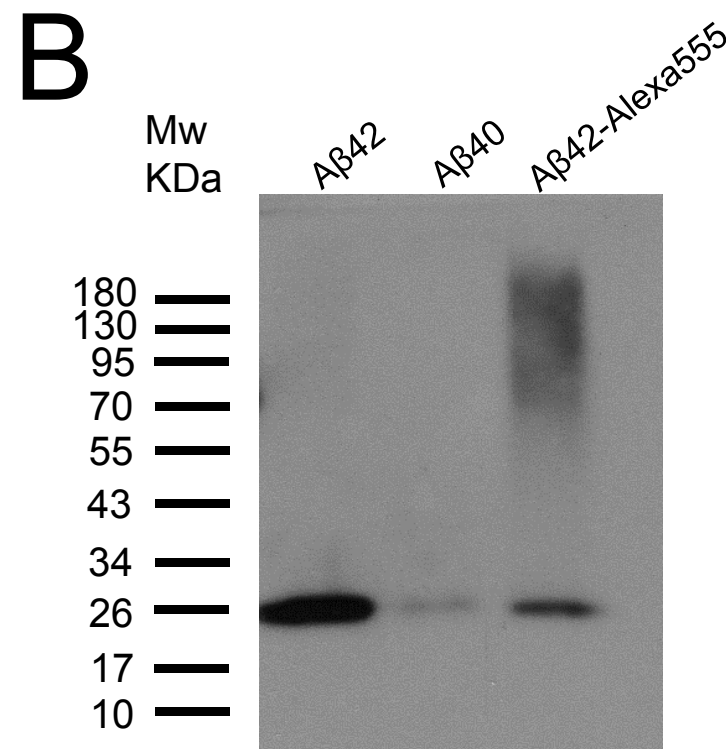

Supplementary Fig 1

Supplement: Additional file 1: Figure S1. — Characterization of oligomeric state of Aβ42, Aβ40 and Aβ42-Alexa555. (A) Atomic force microscopy (performed as described in Additonal file 3) images of Aβ42, Aβ40 and Aβ42-Alexa555 samples after 24 h incubation at 4 °C visualized on graphite. Sizes of field of view for left images are 4×4μm and for right images 1×1μm. Topographic profile for each Aβ sample is presented (measured globule is marked with blue line). All Aβ samples appear primarily as globular structures with following sizes: Aβ42 height 1.56 ± 0.3 nm, diameter at fwhm before deconvolution 75 ± 0.6 nm, diameter at fwhm after deconvolution 9.6 ± 0.1 nm; Aβ40 height 0.9 ± 0.1 nm, diameter at fwhm before deconvolution 43 ± 0.3 nm, diameter at fwhm after deconvolution 7.3 ± 0.1 nm; Aβ42-Alexa555 height 1.46 ± 0.2 nm, diameter at fwhm before deconvolution 68 ± 0.6 nm, diameter at fwhm after deconvolution 8.9 ± 0.1 nm. (B) Supernatant fractions of Aβ42, Aβ40 and Aβ42-Alexa555 preparations were separated on 15 % Acrylamide/Bis SDS gel and analyzed by Western blotting with anti-Aβ 6E10 monolconal antibodies. (PDF 375 kb) [file 13024_2015_34_MOESM1_ESM.pdf]

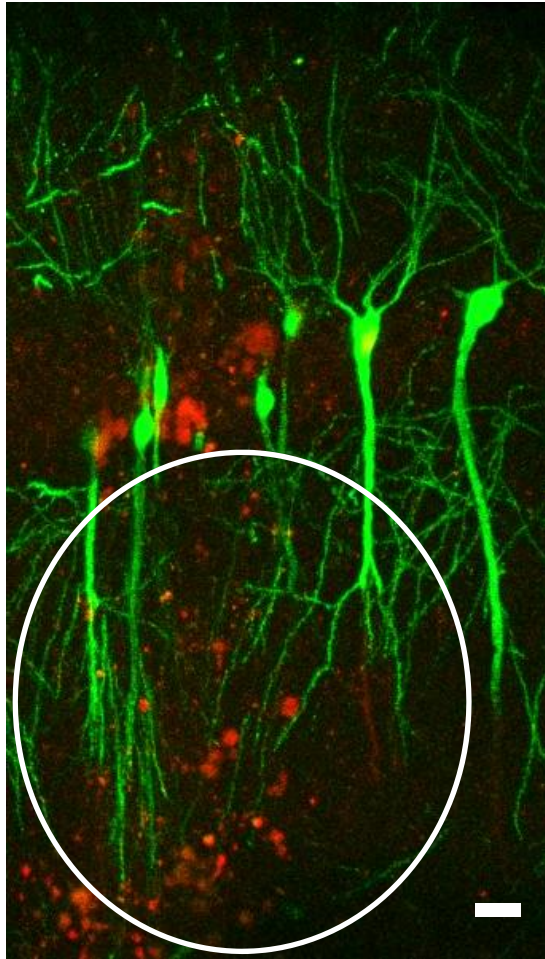

Supplementary Fig 2

Supplement: Additional file 2: Figure S2. — Visualization of hippocampal neurons injected with Alexa-555 labelled Aβ42. Low-magnification image of CA1 hippocampal area from 3.5 months old Thy1-GFP line M mouse is presented. Image is taken six weeks after injection. CA1 neurons expressing GFP protein are shown in green, signals from Alexa-555 labeled Aβ42 are shown in red. The specific dendritic segment where the data on spine density and shape were obtained is marked with white circle. Scale bar corresponds to 20 μm. (PDF 51 kb) [file 13024_2015_34_MOESM2_ESM.pdf]
