# Supplementary material for: STIM2 protects hippocampal mushroom spines from amyloid synaptotoxicity
Source: Mol Neurodegener. 2015 Aug 15;10:37. doi: 10.1186/s13024-015-0034-7 (PMC4536802; doi:10.1186/s13024-015-0034-7)
Supplement: Additional file 3: — Supplementary experimental procedure. Atomic force microscopy. Imaging was performed on commercial SPM Solver P47-Pro atomic force microscope with NSG 11 probe (Nt-MDT Co., Zelenograd, Moscow, Russia). Images were taken in air using tapping mode on highly ordered pyrolytic graphite (Nt-MDT Co., Zelenograd, Moscow, Russia). The surface of graphite was carefully rinsed with deionized water and gently dried under a N2 stream. NovaRC1 operating software version 850 was used to acquire the data images, Gwyddion and SPIP software were used to render the data and perform the analyses. The z-height of 9-10 globules from two different areas on the graphite was measured. Particle analysis was performed by choosing a threshold height equivalent to 1/2 the average z-height of the globules. This measures globule diameter at fwhm (full width at half-maximum). To get approximate width of globules geometrical deconvolution model [36] was used, particles were treated as spheres $$ \mathrm{h}=\raisebox{1ex}{$\sqrt{w/2}$}\!\left/ \!\raisebox{-1ex}{$2Rt$}\right. $$h=w/22Rt where h is the real width of the structure, w is the width or diameter observed in the AFM image, and Rt is the tip apex radius (in our case it is around 10 nm). (DOCX 12 kb) [file 13024_2015_34_MOESM3_ESM.docx]

**Supplementary experimental procedure**

## Atomic force microscopy

Imaging was performed on commercial SPM Solver P47-Pro atomic force microscope with NSG 11 probe (Nt-MDT Co., Zelenograd, Moscow, Russia). Images were taken in air using tapping mode on highly ordered pyrolytic graphite (Nt-MDT Co., Zelenograd, Moscow, Russia). The surface of graphite was carefully rinsed with deionized water and gently dried under a N_2_ stream. NovaRC1 operating software version 850 was used to acquire the data images, Gwyddion and SPIP software were used to render the data and perform the analyses. The z-height of 9-10 globules from two different areas on the graphite was measured. Particle analysis was performed by choosing a threshold height equivalent to 1/2 the average z-height of the globules. This measures globule diameter at fwhm (full width at half-maximum). To get approximate width of globules geometrical deconvolution model [36] was used, particles were treated as spheres

$$h =\frac{\sqrt{w/2}}{2Rt}$$

where h is the real width of the structure, w is the width or diameter observed in the AFM image, and Rt is the tip apex radius (in our case it is around 10 nm).
